# Inhibition of Lymphangiogenesis: A Protective Role of microRNA 146a-5p in Breast Cancer

**DOI:** 10.1155/2024/7813083

**Published:** 2024-08-21

**Authors:** Wenlong Liang, Haoran Wang, Baiyang Fu, Yuan Song, Zheng Zhang, Xin Liu, Yujia Lin, Jianguo Zhang

**Affiliations:** ^1^ Department of Breast Surgery Second Affiliated Hospital of Harbin Medical University, Harbin, China; ^2^ Department of Hepatobiliary Surgery Second Affiliated Hospital of Harbin Medical University, Harbin, China

## Abstract

Breast cancer is the leading cause of death and morbidity among women. A major challenge for clinical management of breast cancer is the dissemination of breast cancer cells from the primary tumor site via lymphatic drainage, resulting in metastatic tumor spread. Recent studies have found that high expression of the microRNA miR-146a-5p is associated with better survival outcomes for breast cancer patients. However, the mechanisms for this prognostic benefit are not fully elucidated, including whether or not miR-146a-5p plays a role in suppression of lymphatic dissemination. In this study, we investigated the role and uncovered functional mechanisms of miR-146a-5p in breast cancer. We found that high expression of miR-146a-5p is associated with better clinical outcomes, specifically in the patients with N0 breast cancer. In culture, miR-146a-5p overexpression in MCF-7 breast cancer cells suppressed cell migration and lymphangiogenesis in lymphatic endothelial cells. When implanted in the mammary fat pad of mice, we observed that miR-146a-5p overexpressing MCF-7 suppressed lymphatic dissemination but had no effect on tumor progression in the primary site. This suppression was associated with fewer disseminated cancer cells and reduced lymphangiogenesis in the draining and distal lymph nodes. In conclusion, these results suggest that miR-146a-5p can exhibit a protective role against breast cancer metastasis, and it can be a therapeutic target for breast cancer.

## 1. Introduction

Breast cancer is one of the most common cancers in women worldwide [1] and is the second most common cancer-related death in women. The Centers for Disease Control and Prevention (CDC) report that there are around 264,000 newly diagnosed cases of breast cancer and approximately 42,000 breast cancer-related deaths each year. One major factor contributing to this high mortality is metastatic tumor spreading through lymphatic vessels [2]. Consequently, lymphatic metastasis has become one of the most important prognostic factors for breast cancer [3]. It is reported by the American Cancer Society that the 5-year relative survival of localized breast cancer is about 99%, but the rate dramatically reduces to 86% and 29% for patients with regional and distant lymph nodes metastasis, respectively. Many studies have indicated that the breast cancer cells can induce remodeling of the lymphatic vessels and regional lymphatic network in sentinel and distal lymph nodes [4]. However, the genes that control lymphangiogenesis in breast cancer are not fully appreciated nor understood. For instance, contradictory findings have suggested that lymphangiogenesis is related with vascular endothelial growth factor receptor-3 (VEGFR-3), vascular endothelial growth factor-C and -D (VEGF-C and VEGF-D), whereas other studies stipulated no correlation between these genes and lymphangiogenesis [5].

MicroRNAs (miRNAs) are a class of noncoding RNAs that play a role in regulating gene expression. It has been reported that miRNAs are involved in developmental lymphangiogenesis [6] and a regulatory role of miRNAs in lymphangiogenesis and angiogenesis has been studied in inflammation and cancer models [7–10]. The mechanism of miRNA regulation of lymphangiogenesis is complex. For example, Jian et al. found that miRNA-126a directs lymphangiogenesis through upregulation of Cxcl12a and Flt4 signaling in zebrafish [11]. Likewise, Stephanie et al. found that miR526b and miR655 upregulated lymphatic vessel endothelial hyaluronan receptor-1 (Lyve1) in breast cancer samples [9]. The miR-146a-5p was recognized as a biomarker in diverse cancers in meta-analysis and bioinformatics analysis [12]. However, whether it is related with lymphangiogenesis of breast cancer or not is unknown.

In this investigation, we studied the positive association between miRNA-146a expression and good prognosis in clinics and further explored its mechanistic role in lymphangiogenesis using *in vitro* and *in vivo* models of breast cancer. These results identify that miRNA-146a as a potentially new marker for breast cancer prognosis and target for biological therapy.

## 2. Results

**Figure 1:**
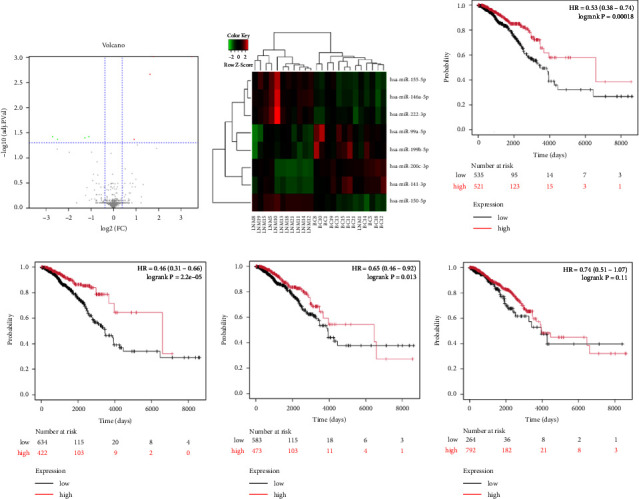
Identification of miRNAs in breast cancer patients with lymph node metastases. (a) Volcano plot of differentially expressed miRNAs in breast cancer patients with or without lymph node metastases. (b) Heatmap depicting the expression of statistically significant differentially expressed miRNAs between lymph node nonmetastasis groups (LNM) and breast cancer with lymph node metastasis group (BC); (c–f) Kaplan–Meier survival curve of patients with high (red) and low expression (black) of hsa-miR-146a-5p, hsa-miR-150-5p, hsa-miR-155-5p, and hsa-miR-222-3p. The *p* value was determined using a log rank test and is displayed on the graph.

**Figure 2:**
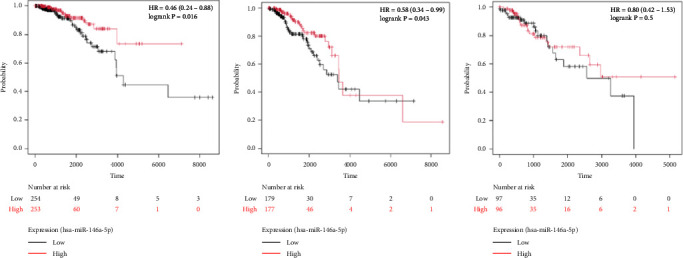
hsa-miRNA-5p associates with better prognosis early stage breast cancer. Kaplan–Meier survival curves comparing breast cancer patients with high expression of hsa-miRNA-5p (red) and low expression of hsa-miRNA-5p (black) at N0 (a), N1 (b), and N2-N3 (c).

To identify miRNAs related with lymph node metastasis, we analyzed miRNA sequencing data from breast cancer patients available from GSE148846 and GSE100453. We classified the patient information into two groups based on lymph node metastasis. Using the bioinformatic analysis, we found that hsa-miR-150-5p, hsa-miR-146a-5p, hsa-miR-155-5p, and hsa-miR-222-3p were upregulated in lymph node nonmetastasis patients (Figures 1(a) and 1(b)). We listed these genes in Table 1. To determine the prognostic consequences of these miRNAs in breast cancer patients, 1056 breast cancer cases were selected and separated into two groups stratified by the expression level. Survival analysis demonstrated that patients with higher expressional levels of hsa-mir-146a, hsa-mir-150-5p, and hsa-mir-155-5p were positively associated with better survival (HR of 0.53, 0.46, and 0.65, respectively; *p* value ≤0.01 respectively; Figures 1(c), 1(d), and 1(e)). No significant difference in survival was observed between low expressers and high expressers of hsa-mir-222-3p (*p* value = 0.11, Figure 1(f)). Based on recent discoveries, we prioritized hsa-miR-146a-5p with a focus on its role in the prognosis of breast cancer, specifically on variant stages (based on clinical TNM staging system). Survival analysis demonstrated that patients with high expression of hsa-miR-146A-5p survive significantly longer (*p*=0.0016, HR = 0.46) when compared with patients with low expression (Figure 2(a)) in N0 stage. In N1 and N2∼3 stages, no differences were observed in hsa-miR-146a-5p expression (Figures 2(b) and 2(c)).

**Figure 3:**
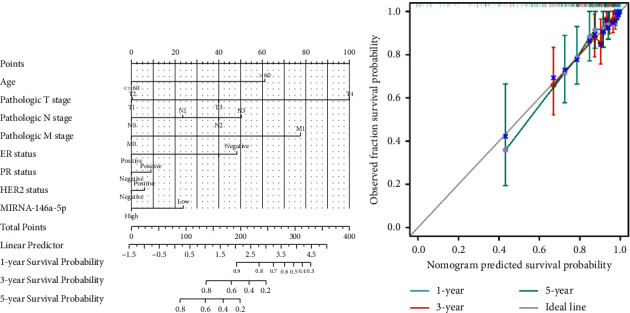
Establishment and assessment of the nomogram model. (a) Nomogram based on clinical characteristic and risk score in HCC patients at 1, 3, and 5 years. (b) Calibration plot of overall survival at 1, 3, and 5 years.

In order to further determine the risk degree and prognosis degree of patients, we combined the expression of miRNA-146a-5p and clinical characteristics of patients with breast cancer in TCGA database to construct the nomogram. On the basis of multivariate regression analysis of clinical information of patients in the TCGA cohort, the overall score was finally calculated to predict the 1-year, 3-year, and 5-year survival rates of patients with breast cancer (Figure 3(a)). To further determine the accuracy of the model in predicting the prognosis, we plotted the correction curve of the model to reflect the degree of agreement between the predicted risk and the actual risk. In the correction curve shown below (Figure 3(b)), we can see that the lines of the prognostic nomogram model are basically well fitted to the diagonal, indicating that the prognostic nomogram had good predictive ability.

**Figure 4:**
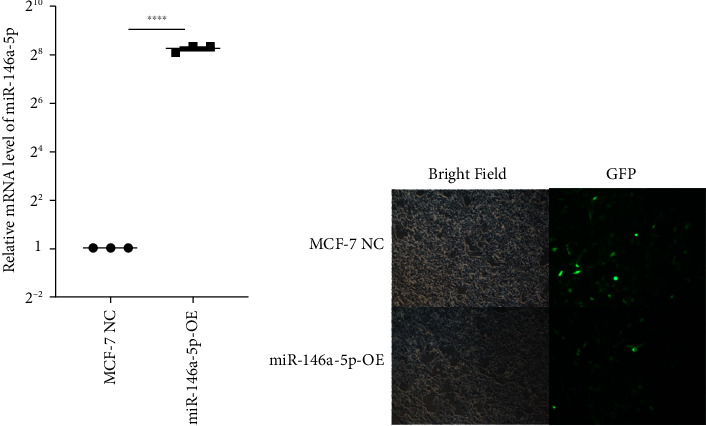
Construction of miR-146a-5p overexpressing MCF-7 cells. (a) Transcription level of miR-146a-5p in MCF-7 NC and miR-146a-5p-OE cells, respectively. (b) Bright-field and fluorescence images of MCF-7 NC and miR-146a-5p-OE cells. ^∗∗∗∗^means *p* < 0.001.

To study the role of hsa-miR-146a-5p *in vitro*, we utilized human MCF-7 cells, an estrogen and progesterone receptor positive invasive ductal/breast carcinoma. We next constructed an overexpression of hsa-miR-146a-5p in MCF-7 (miR-146a-5p-OE) using virus transfection. The transcriptional level of hsa-miR-146a-5p was significantly increased in miR-146a-5p-OE MFC-7 cells when compared with MCF-7 NC (*p* < 0.0001, Figure 4(a)). Additionally, we observed no morphological differences between MCF-7 NC and miR-146-5p-OE (Figure 4(b)).

**Figure 5:**
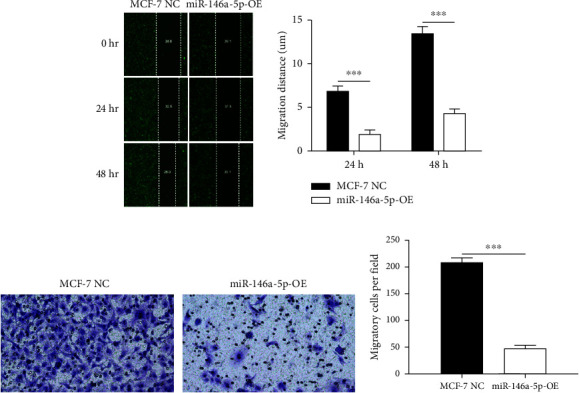
Overexpression of miR-146a-5p inhibits breast cancer cell migration. (a) Fluorescence images of GFP + MCF-7 NC and miR-146a-5p-OE cell migrations in a scratch assay. (b) Quantification of migration distance of MCF-7 NC and miR-146a-5p-OE after 24 and 48 hours. (c) Crystal violet staining images of MCF-7 NC and miR-146a-5p-OE cell migration in a Transwell migration assay. (d) Quantification of the number of cells migrated, per field of view. ^∗∗∗^*p* < 0.001.

**Figure 6:**
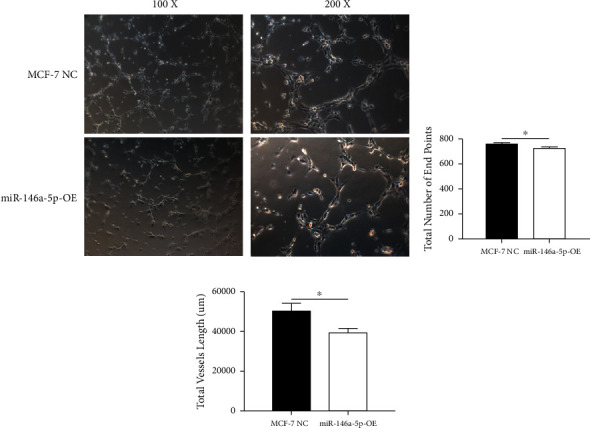
Secreted factors from miR-146a-5p overexpressing cells inhibit production of lymphatic vessels (a) quantification of vessel formation by HLECs cocultured with the supernatant from MCF-7 NC and miR-146a-5p-OE. Graphs show the number of branch ends (b) and vessel lengths (c) ^∗^*p* < 0.05.

Given its predicted role in facilitating lymph node metastasis, we first evaluated the effects of hsa-miR-146a-5p overexpression on cell migration using a classical scratch migration assay (Figures 5(a) and 5(b)) and Transwell migration assay (Figures 5(c) and 5(d)). In scratch migration assays, MCF-7 NC migrated an average distance of 6.8333 *µ*m and 13.4000 *µ*m, at 24 hours and 48 hours, respectively, while miR-146A-5p-OE cells migrated with average distances of 1.9000 *µ*m and 4.2667 *µ*m, respectively (*p*=0.0004 and 0.0001 when compared with MCF-7 NC at 24 hours and 48 hours, respectively). In Transwell migration assays, we found that on average, 208 MCF-7 NC migrated versus only 47 cells of miR-146a-5p-OE (*p* < 0.0001). Next, to determine whether the expression of hsa-miR-146a-5p can affect the production of lymph vessels, we exposed cell culture supernatant from MCF-7 NC and miR-146a-5p-OE to HELS cells (Figure 6(a)). End point lymph vessel formation induced by culture supernatant from miR-146a-5p-OE cells was significantly less than that induced by supernatant from MCF-7 NC cells (723.3333 vs 759.0000; *p*=0.0280) (Figure 6(b)). Similarly, the total vessel length induced by supernatant from miR-146a-5p-OE was also significantly smaller from that induced by supernatant from MCF-7 NC (39331 *µ*m vs 50263 *µ*m; *p*=0.0161) (Figure 6(c)).

**Figure 7:**
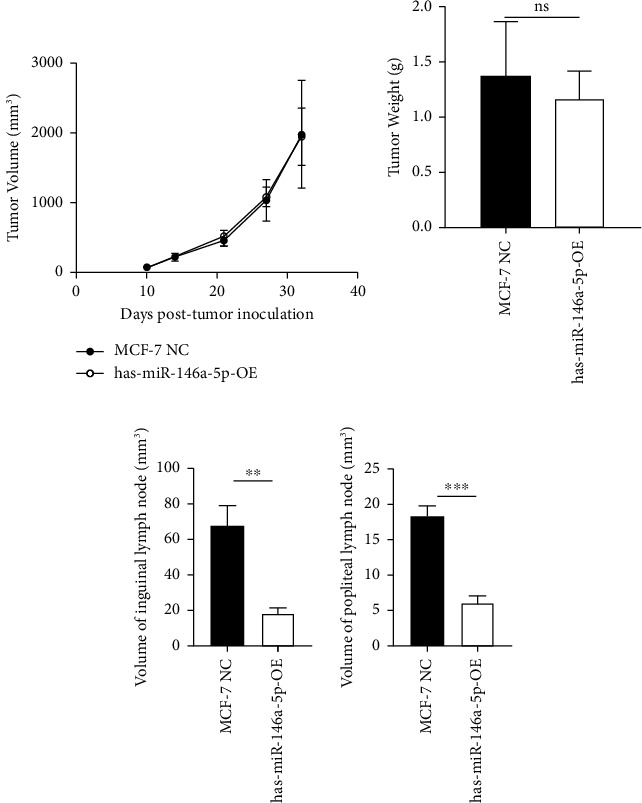
miR-146a-5p inhibits metastasis to the draining and distal lymph nodes. (a) Volume of MCF-7 NC and miR-146a-5p-OE tumors in the mammary fat pad (measured by caliper). (b) Weight of mammary tumors on day 32. (c, d) Volume of inguinal and popliteal lymph nodes from mice bearing MCF-7 NC and miR-146a-5p-OE tumors. n.s.: no significance; ^∗∗^*p* < 0.01; ^∗∗∗^*p* < 0.001.

**Figure 8:**
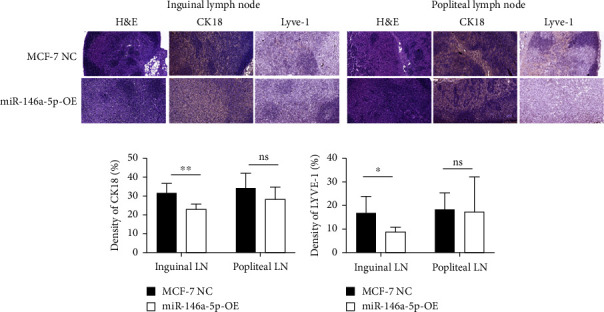
Pathophysiology of inguinal and popliteal lymph nodes in miR-146a-5p overexpressing metastasis. (a) Representative images of core lymph nodes stained with H&E, CK18, and Lyve-1. (b, c) Quantification of CK18 and Lyve-1 staining density in inguinal and popliteal lymph nodes, n.s.: no significance; ^∗^*p* < 0.05; ^∗∗^*p* < 0.01.

To understand the role of miR-146a-5p *in vivo*, we inoculated MCF-7 cells overexpressing miR-146a-5p or control cells in the mammary fat pad of mice and monitored tumor growth over a period of 35 days. The average volume of MCF-7 NC tumors was 84.70 mm^3^, 220.18 mm^3^, 460.85 mm^3^, 1036.52 mm^3^, and 1988.35 mm^3^ on days 10, 13, 21, 28, and 35, respectively, while the volume of miR-146a-5p-OE tumors was 73.14 mm^3^, 221.15 mm^3^, 524.00 mm^3^, 1084.78 mm^3^, and 1952.23 mm^3^, respectively (Figure 7(a), *p* > 0.05). Similarly, there was no difference in tumor weight between MCF-7 NC and miR-146a-5p-OE on day 35 (Figure 7(b), *p* > 0.05). However, the volume of inguinal and popliteal lymph nodes (18.45 mm^3^ and 18.31 mm^3^) from miR-146a-5p-OE tumors was significantly smaller than those from MCF-7 NC tumors (67.56 mm^3^ and 6.145 mm^3^; *p*=0.0039 and *p*=0.0001, respectively) (Figures 7(c) and 7(d)). We hypothesized that this difference was due to fewer cancer cells draining into the lymph nodes in miR-146a-5p-OE tumors and therefore evaluated the expression of CK18 in lymph node tissue sections to quantify metastasizing human cancer cells (Figure 8(a)). In the inguinal and popliteal lymph nodes, the density of CK18 staining was 31.8% and 23.0% in MCF-7 NC tumors, while those in miR-146a-5p-OE tumors were 23.0% and 28.4%, respectively (Figure 8(b), *p*=0.0085 and *p*=0.2361, respectively). Additionally, to characterize pathological changes in lymphangiogenesis, we performed immunohistochemical staining for Lyve-1. The density of Lyve-1 staining was 16.8% and 18.2% in inguinal and popliteal lymph nodes in MCF-7 NC tumors, compared to 8.8% and 17.2% in miR-146a-5p-OE tumors, respectively (*p*=0.0401 and *p*=0.8964, respectively, Figure 8(c)).

## 3. Discussion

MicroRNAs play an important role in the diagnosis and treatment of breast cancer. Previous studies have found that the miR-146a-5p could act as a tumor protective gene [12, 13], but no further studies have been done on lymphatic metastasis. In this study, we found the high expression of miR-146a-5p in the nonlymphatic samples in breast cancer by bioinformatic analysis and validated the role of inhibition lymphangiogenesis in experimental analysis. We also combined the expression of miR-146a-5p with clinical characteristics of patients to predict the prognosis in breast cancer.

The potential mechanisms of hsa-miR-146a-5p in contributing to tumor cell migration and invasion are areas of active investigation. Long et al. found that miR-146a-5p target IL-1R-associated kinase 1 (IRAK1) to inhibit the growth, migration, and invasion of MCF-7 [14]. Liu et al. found that hsa-miR-146a-5p inhibits MDA-MB-231 cell migration by targeting RhoA, which plays an important role in the regulation of cytoskeleton reorganization [15]. Consistently in breast cancer cells, Do et al. found that hsa-miR-146a-5p could suppress NRP2-SEMA3C to regulate cell migration/invastion [16]. In non-small-cell lung cancer (NSCLC) cells, Chen et al. found that hsa-miR-146a-5p could inhibit cell growth and migration and induce apoptosis by targeting EGFR and NF-kB signal pathway [17]. Wang et al. found that hsa-miR-146a-5p inhibited oral squamous cell carcinoma cell migration/invasion by targeting the huntingtin gene [18]. Finally, Afshar-Khamseh et al. have found that hsa-miR-146a-5p downregulated the CXCR4 gene, which has implications for colorectal cancer development and metastasis [19]. These literature studies demonstrated that hsa-miR-146a-5p might play a protective role in breast cancer metastasis. However, the role of miR-146a-5p in other cancer is complicated and varied in different literature studies, which could be due to different cancer types and different cellular sources. A meta-analysis of clinical analysis showed its positive association with better survival in reproductive system cancers (including breast invasive cancer) and digestive system cancers but not other cancers [12]. Furthermore, the intercellular roles of miR-146a-5p are always controversial. For instance, miR-146a-5p from cancer-associated fibroblast was believed to be related with stemness in bladder cancer [20] and metastasis of colorectal cancer [21]. Sponging miR-146a-5p by IL-8 from GC-MSCs can promote gastric cancer progression [22].

To study the MCF-7 miR-146-5p-OE metastasis, we utilized a mammary fat pad tumor model. At the primary site, we observed that miR-146a-5p-OE tumors were similar in size to controls but had different burden of tumor at the draining and distal lymph nodes (lymphatic metastasis). This system mimicked the early stage of clinical breast cancer and demonstrated that hsa-miR-146a-5p played an important role in lymphatic metastasis. We additionally observed that miR-146a-5p-OE had smaller sizes of draining inguinal lymph nodes, as well as less CK18+ metastatic cancer cells. When studying the pathology of distant metastasis in popliteal lymph nodes, we observed larger nodes with a trend towards containing more metastatic cancer cells. These enlarged lymph nodes could indicate an early immune response associated with the premetastatic cascade. MCF-7 miR-146a-5p-OE tumors had fewer cancer cells metastasis, which was associated with less Lyve-1 expression (less lymphangiogenesis) in draining lymph nodes. The detailed mechanism of how hsa-miR-146a-5p influences lymphangiogenesis remains to be elucidated but may be related with inhibition of NF-kb. For instance, in THP-1 cells, it has been reported that hsa-miR-146a-5p can inhibit NF-kb activation [23], and the NF-kb activation during inflammation can induce lymphangiogenesis [24]. Furthermore, the expression of some downstream gene including MMP9, MMP2, and CXCR4 are repressed by has-miR-146a restoration [25]. These genes are believed to play a positive role in lymphangiogenesis [26, 27], as well as the progression and metastasis of breast cancers and other malignancies [28, 29]. These studies indicate that miR-146-5p expression may modulate activation of the NF-kb signaling pathway, which inhibits lymphangiogenesis gene transcription, and results in reduced lymphatic metastasis.

There are some limitations in the present study. As described previously, cancer stage/metastasis and comorbidities are determinants of prognosis. Whether the expression of miR-146a-5p is stable during breast cancer progression remains unclear. Future investigations should analyzed miR-146a-5p in larger clinical cohorts to confirm our findings and determine relationships between miR-146a-5p and breast cancer subtypes. Another limitation is that we did not identify genes that are regulated by overexpression of miR-146a-5p. RNA-sequencing of miR-146-5p overexpressing cells could enable insight into the genes and pathways that drive this phenotype. As an alternative and complementary approach, miR-146a-5p could be knocked down in breast cancer that endogenously expresses the miRNA. Changes in transcriptional and functional phenotypes could be evaluated by RNA-sequencing and *in vivo* evaluation of metastasis.

In summary, we discovered that high expression of hsa-miR-146a-5p was associated with better prognosis, specifically in patients with N0 stage breast cancer. Utilizing a mouse model that mimicked early stage breast cancer metastasis, we found that hsa-miR-146a-5p was associated with fewer metastasizing cancer cells, through inhibition of lymphangiogenesis. This study provides insight into a new role for hsa-miR-146a-5p in tumor metastasis and identifies hsa-miR-146a-5p as a new biomarker for prognosis assessment and a target for breast cancer new treatments (new medications screening, RNA interference, or other biological therapy) in the future.

## 4. Methods and Materials

### 4.1. Clinical Data and Bioinformatics Analysis

MicroRNA sequencing of breast cancer patients with lymph node metastasis (LNM) or without lymph node metastasis (BC) were downloaded from the GEO database (GSE148846 and GSE100453). MicroRNA expression profiling of paired primary and lymph node of 24 metastatic breast cancer patients was available between these two datasets. The Limma package and ggplot2 package in R were used to identify differentially expressed genes and for visualization. The transcriptional levels of miRNA were analyzed and visualized using volcano plots and heatmaps. Clinical breast cancer cases (1056 samples with complete clinical and gene expression profile data) were downloaded from TCGA database and divided into high expressers and low expressers (miRNA expression level greater than or less than 50) for survival analyses. We used the R software package RMS to integrate the survival time, survival status, and clinical characteristics data of the samples in TCGA, combined with the expression of miRNA-146a-5p, and established a prognostic nomogram on the basis of multifactor regression to predict the survival prognosis of patients with breast cancer. Meanwhile, a calibration curve was drawn to test the accuracy of the prognostic nomogram.

### 4.2. Cell Lines

Human breast cancer cell lines MCF-7, human lymphatic endothelial cells (HLECs), were purchased from iCellbioscience (Cat# iCell-h067 and iCell-h129, respectively). DH5*α* competent cells and 293T cells were purchased from ThermoFisher (Cat #: 18265017) and Sigma (Cat# 12022001-1VL), respectively. All cells were cultured in RPMI 1640 (Corning #10-040-CVB) or DMEM (Corning #R10-017-CV) with 10% Gibco FBS (Invitrogen # 16000−044) in a 37°C incubator with 5% CO_2_.

### 4.3. Transcription Comparison and High-Expression Mutant Construction

The human MIR146A sequence was obtained from NIH (Gene ID: 406938) and amplified by PCR. The double-stranded DNA was ligated into the LVO-114 vector (plasmid hU6-MCS-CMV-EGFP, mimics from YBR BIOSCIRES Cat# BR-V108) digested by Age I enzyme (ThermoFisher ER1461) to generate the MIR146A containing vector. The vector was transformed into competent *E coli* DH5a cells in AMP resistance (100 *μ*g/ml) LB agar medium, and the single clone containing MIR146A expressing vector was screened with PCR and further confirm with sequenced, while the no-treatment LVO-114 vector was employed as the empty vector. To pack the recombinant lentiviral vector, the plasmid was abstracted and purified with QIAprep Spin Miniprep Kit (QIAGEN Cat#27140) and further transfected into the 293T cells with Lipofectamine2000 (ThermoFisher Cat#11668027). The high purity lentiviral concentrated liquid was collected from the supernatant after 48 hours of culture. After 72 hours of coculture of MCF-7 cells and lentiviral vector (MIR146A expressing vector and empty vector) in RPMI with 10% FBS and puromycin (1 *μ*g/ml), the stable transfectants were selected.

Expression levels were determined using RT-qPCR. Total RNA was extracted from cells by using TRIzol™ Plus RNA Purification Kit (ThermoFisher Cat # 12183555). cDNA was synthesized from mRNA using the TransScript® One-Step gDNA Removal and cDNA Synthesis SuperMix (TRANS, Cat # AT311−02), and miRNA reverse transcription was performed using TaqMan™ Advanced miRNA cDNA Synthesis Kit (ThermoFisher Cat # A28007). The data were calibrated to U6 and calculated using the 2^−ΔΔCT^ method. Primer sequences are listed as follows: hsa-miR-146a-F: GCGCCGTGAGAACTGAATTC; hsa-miR-146a-R: GTGCAGGGTCCGAGGT; hsa-miR-146a-RT: GTCGTATCCAGTGCAGGGTCCGAGGTATTCGCACTGGATACGACAACCCATG; U6–F: CTCGCTTCGGCAGCACA; U6-R: AACGCTTCACGAATTTGCGT.

### 4.4. *In Vitro* Scratch Assay and Transwell Assay

Scratch assay was performed per established protocols [30]. Cells were seeded at a density of 5e4 cells per well. After 8 hours, a confluent monolayer (∼90% confluence) was formed. A 5 *µ*m scratch on the monolayer was made using an AutoScratch Wound Making Tool (GeneSci Cat#YBR1347). Following 24 hrs and 48 hrs of culture in media containing 0.5% FBS, the migration of cells were identified and analyzed by Cellomics.

Transwell assays were performed as previously described [31]. 600 *µ*L of the medium supplemented with 30% FBS was added to the lower compartment, and 1e5 cells were added into the upper compartment. After 24 hours, nonmigratory cells in the upper chamber were removed and the number of migratory cells beneath the permeable membrane was calculated.

### 4.5. Lymphangiogenesis Assay

*In vitro* lymphangiogenesis assay was performed per established protocols [32]. Supernatant/culture medium from each cell line was collected and concentrated 10x (Amicon Ultra centrifugal filter REF# UFC903024 from Millipore Billerica MA, USA) when the confluence of each cell line reached 100%, and the medium was diluted with the endothelial cell growth medium (sigma cat#211–500) with a ratio of 1 : 10.200 *μ*l of the mixed medium were added to 500 cells of HLECs per wells (on a 12 well plate). After 12 hours of coculture, the connections between HLECs form vessel-like structures. The new-formed vessels were analyzed under an inverted microscope. The endpoints and lengths of these vessel structures per field of view were calculated by using AngioTool.

### 4.6. Mammary Fat Pad Breast Cancer Model

4∼6-week old female BALB/c mice (5 mice in each group) were utilized for *in vivo* experiments. 1*E* + 7 MCF-7 NC and miR-146a-5p-OE cells suspended in 200 *µ*L of PBS were injected into the breast adipose tissue near the rear leg. Animals were carefully monitored daily for tumor size and euthanized on day 32. Tumor tissue, draining inguinal lymph nodes, and distant metastasis popliteal lymph nodes were harvested and preserved in 10% formalin for further histopathological examination. The tumor volumes were estimated based on the measurement of diameters taken on days 10, 14, 21, 28, and 32. Tumor weight was measured after the tumor removal from euthanized mice on day 32.

### 4.7. Pathology (H&E and IHC Staining)

For H&E staining, 5 *µ*m tissue (tumor and lymph nodes) sections were affixed to microscope slides. The slides were deparaffinized by 3 changes of xylene for 8 min and rehydrated in 3 changes of 100% ethanol (5 minutes each), followed by 75% ethanol (3 minutes). Next, the slides were stained with hematoxylin solution for three mins, washed with tap water for 2 minutes, and counterstained in eosin solution for 25 seconds. The stained slides were washed with tap water for 2 minutes, dehydrated through 95% ethanol (20 seconds) and 2 changes of 100% ethanol (2 minutes each), and cleared with 2 changes of xylene (2 minutes each). Finally, the slides were mounted with a neutral mounting medium.

For IHC, the slides were handled, deparaffinized, and rehydrated as described above. For antigen retrieval, slides were inserted into a metal chamber filled with Tri-EDTA buffer (10 mM Tris base, 1 mM EDTA solution, 0.05% Tween 20, pH 9.0) and processed in pressure cooker at 100°C for 15 mins and then cooled down to room temperature. After rinsing with PBST buffer, endogenous peroxidases were blocked by incubation with 3% hydrogen peroxide for 5 minutes. Secondary antibodies were further blocked with 5% goat serum for 15 minutes. Slides were then stained with primary antibodies (CK18 Bioss bsm-60699R and Lyve-1 Abcam ab218535) followed by secondary (goat anti-rabbit Ab Abcam ab97080). After washing with PBST, slides were developed with chromogen 3, 3′-diaminobenzidine (DAB). Finally, slides were counterstained with hematoxylin (5 mins), dehydrated with ethanol, cleared with xylene, and mounted with mounting medium for further analysis. Quantification of CK18 was expressed as a percentage of positive cell number over total cell number, and quantification of Lyve-1 was expressed as the density of positive signal over the area of whole lymph node.

### 4.8. Statistical Analysis

Survival analysis was performed using the R statistical programming language. All other statistical analyses were carried out in GraphPad Prism 8.3.0. Transcriptional analysis was calibrated to a housekeeping gene which was expressed in ΔCT values and log_2_-transformed expression. Other data were presented as the mean ± SEM. Transcriptional analysis of miR-146a-5p, migration distance, migratory cell number, lymphatic vessel endpoints and length, tumor weight, lymph nodes sizes, and CK18 and Lyve-1 density were evaluated using unpaired student's *t*-test. A *p* value <0.05 was considered statistically significant.

## Figures and Tables

**Table 1:** The different expression genes between the two groups.

Gene symbol	logFC	*p* value	Adj. *p* value
hsa-miR-150-5p	3.469199	3.89*E* − 07	0.000953
hsa-miR-200c-3p	−2.70505	7.35*E* − 05	0.037731
hsa-miR-141-3p	−2.48341	0.000124	0.042628
hsa-miR-146a-5p	1.774138	7.53*E* − 07	0.000953
hsa-miR-155-5p	1.615275	2.55*E* − 06	0.002149
hsa-miR-99a-5p	−1.27591	9.43*E* − 05	0.039798
hsa-miR-199b-5p	−1.09874	7.45*E* − 05	0.037731
hsa-miR-222-3p	0.914785	0.000135	0.042628

## Data Availability

Publicly available datasets analyzed in this study can be found in the GEO database.
